# Draft Genome Sequence of Strain LSUCC0135, an Early Diverging Member of the Order *Methylophilales* in the Phylum *Betaproteobacteria*

**DOI:** 10.1128/genomeA.01231-16

**Published:** 2016-11-03

**Authors:** V. Celeste Lanclos, Michael W. Henson, David M. Pitre, J. Cameron Thrash

**Affiliations:** Department of Biological Sciences, Louisiana State University, Baton Rouge, Louisiana, USA

## Abstract

We present the draft genome of *Methylophilales* sp. strain LSUCC0135, isolated using high-throughput dilution-to-extinction culturing methods from the coast of Freshwater City, Louisiana, USA. The genome indicates metabolic flexibility for differing oxygen concentrations and electron donors.

## GENOME ANNOUNCEMENT

*Methylophilales* sp. strain LSUCC0135 was isolated using high-throughput dilution-to-extinction culturing methods from seawater collected from the coast of Freshwater City, Louisiana, USA (1). Operational taxonomic units (OTUs) matching LSUCC0135 were found in four of seven sampling sites with waters of varying salinity ranging from fresh to salt (1.72 to 22.16 ppt), and temperatures ranging from ~9°C to ~30°C (1). It was the 79th most abundant OTU from its collection site. Phylogenetic placement showed it to be an early diverging lineage associated with the *Methylophilales* (1), with *Methylopumilus planktonicus* (2) as the closest cultured representative according to BLAST (96% identity, accession number NZ_LN827929.1). The family *Methylophilaceae* currently comprises four recognized genera: *Methylobacillus*, *Methylophilus*, *Methylovorous*, and *Methylotenera* (3, 4), as well as the important OM43 marine clade (5, 6). LSUCC0135, however, did not obviously associate with any of these, and we therefore designated it as a candidate for genomic analysis.

We extracted DNA for genome sequencing using a PowerWater kit (MoBio Laboratories, Carlsbad, CA, USA) following the manufacturer’s protocol. Resultant DNA was shotgun-sequenced on an Illumina MiSeq to generate paired-end 250-bp reads at Argonne National Laboratory in Chicago, IL, USA. Sequencing resulted in 238,480 raw reads. We completed genome assembly using the A5-Miseq pipeline with default settings (7) and used CheckM to estimate quality and contamination (8). Annotation of the resultant scaffolds was completed through the Joint Genome Institute IMG/ER website (9).

The draft genome of *Methylophilales* sp. strain LSUCC0135 is 2,106,879 bp and consists of 25 scaffolds with an estimated completion of 99.6% and “contamination” predicted to be less than 1.4%. There are 2,162 predicted genes, 2,105 protein-coding genes, and it has a GC content of 55.2%. The genome contains 44 tRNAs, as well as three 5S and one each of the 16S and 23S rRNA genes. There are also three predicted CRISPR arrays and no predicted motility genes. Members of the *Methylophilaceae* family are known to utilize methanol and methylated amines (3), and we found *mxaLDC* genes for methanol oxidation to formaldehyde in LSUCC0135. The genome codes for glycolysis using the pentose phosphate pathway, fructose metabolism, and phosphoribulokinase and ribulose-1,5-bisphosphate carboxylase, indicating the potential for both heterotrophy and autotrophy. We found a complete assimilatory sulfate reduction pathway and *soxYZ* sulfur oxidation genes. Members of the *ompR* two-component system *phoRB* and the PII gene superfamily likely allow for survival in environments with limited phosphate (10) and nitrogen (11), respectively. LSUCC0135 also contains *coxABC* and *ccoNOQP* cytochrome *c* oxidase genes, suggesting flexibility for life under differing oxygen tensions (12).

### Accession number(s).

Contigs from the assembled genome were deposited into GenBank under the accession number MCAW00000000. The IMG annotation has GOLD Project ID Gp0124971.
